# Comparing the Duration of the Analgesic Effects of Intravenous and Rectal Acetaminophen Following Tonsillectomy in Children

**DOI:** 10.5812/aapm.13175

**Published:** 2014-02-06

**Authors:** Soudabeh Haddadi, Shideh Marzban, Mohammad Seddigh Karami, Abtin Heidarzadeh, Arman Parvizi, Bahram Naderi Nabi

**Affiliations:** 1 Anesthesia Research Center, Guilan University of Medical Sciences, Rasht, Iran

**Keywords:** Acetaminophen, Pain, Postoperative, Child, Hospitalized

## Abstract

**Background:**

Postoperative pain control (especially, after adenotonsillectomy) has a very important effect on recovery time, hospitalization duration, hemodynamic disorders, bleeding, nausea, vomiting and medical costs.

**Objectives:**

The aim of this study was to investigate and compare the effects of intravenous and rectal acetaminophen on controlling post-adenotonsillectomy pain in children, and duration of their analgesic effects.

**Patients and Methods:**

In this randomized double-blinded clinical trial, 96 children aged 4 - 10 years old with ASA physical status I or II who were candidates for adenotonsillectomy surgery in Amir-al-Momenin Hospital, Rasht, Iran were entered into the study and randomly divided into two equal groups. Anesthesia in both groups was induced injecting fentanyl-thiopental and at racurium; afterwards is of lurane was used to maintain anesthesia. After anesthesia induction, one group received intravenous and the other one, rectal acetaminophen, and were later compared based on CHIPPS criteria.

**Results:**

Data analysis indicated a significant relationship between reduction of postoperative pain and the use of intravenous or rectal acetaminophen (P = 0.0001); in group receiving IV acetaminophen, only 10.4% of patients had no pain whereas in group receiving rectal acetaminophen, this number reached 43.8%. Also, on 4 and 6 hour time intervals, pain in rectal acetaminophen receiving group was less than that in IV acetaminophen receiving group (P < 0.05). Demand for additional analgesic medication in rectal acetaminophen receiving group was less than that in IV group (P = 0.0001).

**Conclusions:**

Post-operative pain in rectal acetaminophen group was less than that in intravenous acetaminophen group, and rectal acetaminophen group demanded their first additional analgesic medication later.

## 1. Background

Tonsillectomy with or without adenoidectomy is the most prevalent surgical procedure in children (1), indicated for recurred tonsillitis, peritonsillar abscess, obstructive sleep apnea, and removal of tumoral tissues (2, 3).

This surgery is accompanied by complications like nausea and vomiting, bleeding, and postoperative pain. Pain, if not controlled, is one of the most prevalent complications of tonsillectomy, which especially in children, may result in longer period of recovery and later discharge, eating disorder, and dehydration leading to longer hospitalization duration and continuation of IV fluid intake (4-6).

On the other hand, most patients undergoing this surgery are children, who have lower pain tolerance thresholds and experience earlier restlessness having negative psychological effects on them and their parents (4). In many articles and studies, different medications like opioids, NSAIDS, steroids and acetaminophen have been reviewed for reducing this pain (4). NSAIDs are commonly used for analgesia after tonsillectomy; although, their usage is controversial because of the increased risk of platelet adhesion disorder leading to postoperative bleeding (7).

Steroids such as dexamethasone are prescribed to control nausea and post-tonsillectomy pain, but complications such as increased blood pressure as well as digestive and hormonal disorders might progress (2).

In some studies, the efficacy of lidocaine, morphine, and ketamine spray were compared (8, 9). Efficacy of honey on post-tonsillectomy pain has also been investigated (10).

Acetaminophen is a non-opioid analgesic used to reduce mild to moderate musculoskeletal, osteoarticular, menstrual and post minor-surgery pains and head, and toothache. Its analgesic and antipyretic effects are similar to those of aspirin. However, unlike aspirin, it has no anti-inflammatory effects and does not show many side effects of NASIDs like gastrointestinal disorders, anticoagulant effects, and kidney activity (8).

Oral acetaminophen exhibit sun predictable plasma concentration; also, in patients with inability to swallow for example after tonsillectomy or inpatients suffering from nausea, and vomiting, it is of limited use.

Rectal form of acetaminophen may not be easily accepted and not induce sufficient therapeutic plasma levels. Intravenous acetaminophen makes minor changes in plasma concentration and induces predictable plasma level; thus, it is more easily accepted by patients, has no special complications during injection, and facilitates the intended treatment (1).

On the other hand, acetaminophen seems to be low risk drug with no prevalent side effects. Of course, consuming high and long-term doses may lead to toxicity and liver damage. Its maximum permissible dose is 4 g, administered as 325-500 mg per 4-6 h in adults and 120-240 mg per 4-6 h in children. Acetaminophen suppository is presented with doses of 125 and 325 mg, and its intravenous form is available in 1 g vials with volume of 6.7 mL (11).

Acetaminophen suppository has long been used for post-tonsillectomy pain reduction and development of its intravenous form in recent years has expanded its analgesic and antipyretic consumption. Given acetaminophen suppository's wide use for post-tonsillectomy pain reduction and considering the few studies addressing the IV form effects, this study was developed to compare the two.

## 2. Objectives

Searching the previous articles, we did not find any trial working on administration of rectal or intravenous acetaminophen to relieve post-adenotonsillectomy pain, therefore considering the multiple negative effects of post-operative pain, we decided to compare the duration of their analgesic effects following adenotonsillectomy in Amir-Al-Momenin Academic Hospital, Rasht, Iran.

## 3. Patients and Methods

After writing the proposal, receiving justification register number from Vice-Chancellor of research department of Guilan University of Medical Science, and registering the study in Iranian Randomized Clinical Trial Site (IRCT) under No: 201107301138N8, we started this double–blinded clinical trial . This study was conducted on 96 children aged 4-10 years old with ASA physical status I or II, who were all candidates of elective adenotonsillectomy or adenoidectomy (inclusion criteria) from 1390 to 1391. Exclusion criteria included emergency surgery, identified allergy to studied drugs, history of known and active renal, liver, respiratory or cardiovascular diseases, seizure, neurological or neuromuscular disorders, history of chronic pain, or constant the use of analgesic drugs.

After explanation of study issues and fulfilling the informed consent, patients were divided into two groups randomly. The simple randomization was done through selection of either A (for IV acetaminophen) or B (for rectal acetaminophen) card by each patient.

The study was organized in a double-blinded design (i.e. neither the patient nor the assessor knew about the administered medication); however, only the prescribing person was aware of the prescribed drug in order to take required measures in case of unfavorable medication complications.

After intravenous access line establishment; all the children received normal saline 0.9%, 5-10 cc/kg. All the subjects were monitored using electrocardiography, pulse oximetry, capnography, non-invasive blood pressure measurement, and precordial stethoscope during the surgery.

All the children received 0.01 mg/kg atropine, 2 μg/kg fentanyl, and 0.1 mg/kg dexamethasone as antiemetic. Anesthesia was induced by 3-5 mg/kg sodium thiopental and 0.5 mg/kg at racurium. After endotracheal intubation, anesthesia was maintained by 0.5-0.6% isoflurane, 50% oxygen and 50% NO_2_, and the patients' ventilation was monitored (Tidal Volume = 8-10 cc/kg, RR = 14 breath/min, FiO_2_ = 50%). Surgery was performed by a unique surgical team of Otolaryngologist residents.

After anesthesia induction, groups A and B received intravenous acetaminophen 10 mg/kg (using 1000 mg vials from UNI-PHARMA S.A. Kifissia, Greece) and rectal acetaminophen 15 mg/kg (using 125 and 325 mg suppositoriy from Aburaihan Co. Tehran, Iran), respectively. At the end of surgery and after extubation, the patients were transferred to recovery room in tonsil position and put under ECG/POM/NIBP monitoring while receiving 4 - 5 lit/min oxygen via a facial mask.

Postoperative pain was assessed based on CHIPPS criteria (children and infants postoperative scales) (Table 1) and restlessness was evaluated by a four-point scoring system (1). 

[1- Quiet child (no need for intervention), 2– A child that could be comforted (requiring physical contact with parents), 3- Restless child (crying), 4- Child in aggressive mode (requiring physical control to prevent harming themselves)] (4).

**Table 1.** Children and Infants Postoperative Scales (CHIPPS)

Crying	Points
**No Crying**	0
**Whining**	1
**Screaming**	2
**Facial Expression**	
**Calm**	0
**Grimacing**	1
**Frowning**	2
**Body Condition**	
**Supine**	0
**Variable**	1
**Agonizing**	2
**Posture of Organs**	
**Immobile**	0
**Bending**	1
**Kicking**	2
**Psychomotor Status**	
**Motionless**	0
**Average Motion**	1
**Restless**	2

In case the patients were suffering from the pain in spite of full dose acetaminophen administration (CHIPPS > 4), pethidine (0.5 mg/kg) was injected intravenously.

Finally, collected data were analyzed by statistical software (SPSS ver.16). Chi square and T-test were used for data analysis. P-value was less than 0.05, was considered assignificant. 

## 4. Results

The study was conducted on 96 children aged 4-10 years old who were candidates for adenotonsillectomy surgery from 1390 to 1391 in the Amir-Al- Momenin Academic Hospital. The patients were randomly divided into two groups receiving IV and rectal acetaminophen, and were supervised in recovery room until 24 hours in ward in terms of pain frequency, time of receiving the first additional analgesic medication, and pain score.

Patient characteristics were described in Table 2. 

**Table 2.** Frequency of Demographic Characteristics in Children Undergoing Adenotonsillectomy Surgery

Variable	No. (%)	P value
**Gender**		0.539
Boy	52 (54.2)	
Girl	44 (45.8)	
**Age Group, y**		0.127
Less than 5	11 (11.5)	
5-10	80 (83.3)	
More than 10	5 (5.2)	
Mean ± SD	7.53 ± 1.89	
**Weight, kg**		0.095
Below 25	43 (44.8)	
25-35	33 (34.4)	
Above 35	20 (20.8)	
Mean ± SD	29.05 ± 9.95	
**Patient's Condition Upon Arrival at Recovery**		0.207
Calm	50 (52.1)	
Restless for visiting parents	12 (12.5)	
Crying	28 (29.2)	
Extremely restless and needing physical control	6 (6.2)	

After reviewing the data, no statistically significant relationship was observed between situation of children upon arrival at the recovery and using various forms of acetaminophen (IV or rectal) as analgesic drugs (P = 0.207) (Table 2). 

**Figure 1.**
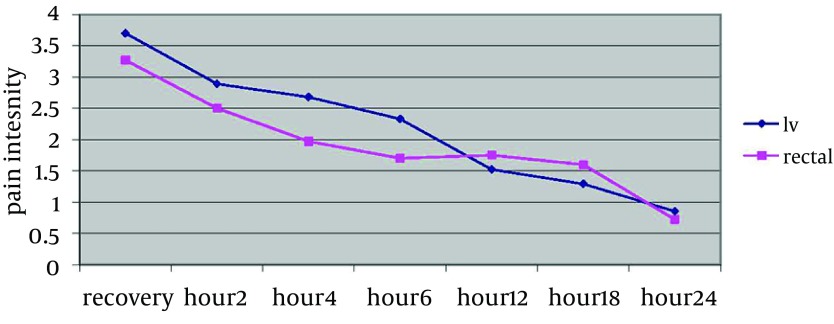
Comparing Trend of Changes in Pain Intensity Based on CHIPPS Criteria in the Studied Time Intervals in Both Groups of Children Using Intravenous or Rectal Acetaminophen. T-test was used to reveal a statistically significant difference between mean of patients' pain intensity on the 4th and 6th hours (P < 0.05); accordingly, in rectal acetaminophen group, it was considerably less than that of the IV acetaminophen group (Table 4).

Comparing trend of changes in patients' pain score based on CHIPPS criteria, a statistically significant relationship was found between mean pain scores at different time intervals (P = 0.0001I Vgroup and P = 0.0001 Rectal group) (Table 3). However, using General Linear Model, it was revealed that the trend of changes in mean pain status at different time intervals was not significantly different among two groups (P = 0.562), and the pain trend of both groups showed a descending state (Figure 1). 

**Table 3.** Comparing Trend of Changes in Pain Status Based on CHIPPS Criteria in the Studied Time Intervals for Two Groups of Children Using Intravenous and Rectal Forms of Acetaminophen as Analgesic ^a^

Group	No.	Mean ± SD	t value	Intra-Group P value
**Intravenous Acetaminophen**			18.12	0.0001
Recovery	48	3.7 ± 2.33		
Until 2nd h	48	2.89 ± 1.37		
Until 4th h	48	2.68 ± 1.5		
Until 6th h	48	2.33 ± 1.61		
Until 12th h	48	1.75 ± 1.6		
Until 18th h	48	1.6 ± 1.28		
24th h	48	0.85 ± 1.09		
**Rectal Acetaminophen**			24.97	0.0001
Recovery	48	3.27 ± 2.06		
Until 2nd h	48	2.5 ± 0.58		
Until 4th h	48	1.97 ± 0.48		
Until 6th h	48	1.7 ± 0.79		
Until 12th h	48	1.52 ± 1.03		
Until 18th h	48	1.29 ± 1.5		
Until 24th h	48	0.72 ± 1.06		

^a^ T value and Intra group P value is 0.762 and 0.562, respectively.

**Table 4.** Comparing Patients' Mean Pain Status Based on CHIPPS Criteria in the Studied Time Intervals in Two Groups of Children Using Intravenous and Rectal Acetaminophen as Analgesic

Time Period	No.	Mean	SD	t value	P value
**Recovery**				0.974	0.333
IV acetaminophen	48	3.7	2.33		
Rectal acetaminophen	48	3.27	2.06		
**Until 2nd h**				1.83	0.071
IV acetaminophen	48	2.89	1.37		
Rectal acetaminophen	48	2.5	0.58		
**Until 4th h**				3.1	0.003
IV acetaminophen	48	2.68	1.5		
Rectal acetaminophen	48	1.97	0.48		
**Until 6th h**				2.4	0.019
IV acetaminophen	48	2.33	1.61		
Rectal acetaminophen	48	1.7	0.79		
**Until 12th h**				8.32	0.408
IV acetaminophen	48	1.75	1.6		
Rectal acetaminophen	48	1.52	1.03		
**Until 18th h**				1.09	0.276
IV acetaminophen	48	1.6	1.28		
Rectal acetaminophen	48	1.29	1.5		
**24th h**				0.567	0.572
IV acetaminophen	48	0.85	1.09		
Rectal acetaminophen	48	0.72	1.06		

Moreover, using the Chi-square test, a significant relationship was determined between pain (demanding medical treatment) after the end of anesthesia (CHIPPS values higher than 4) and using various acetaminophen forms (IV or rectal) (P = 0.0001); accordingly, in IV acetaminophen receiving group, only 10.4% of people were pain free, but in rectal acetaminophen receiving group, this amount reached to 43.8%. Consequently, the use of additional painkillers was higher in IV acetaminophen group; resulting in a statistically significant relationship (P = 0.0001).

Statistical investigations and t-test, revealed that there was no significant relationship between mean pain score of children in the first hour after receiving acetaminophen by the following the operation in both IV and rectal groups showing pain score of higher than 4 (P = 0.452); however, rectal acetaminophen receiving group were administered additional doses of analgesic later than IV group (mean time of 4.96 versus 3.81 h). In terms of age, weight, and gender, there were no statistically significant relationships for the first hour analgesic consumption (Table 5). 

**Table 5.** Comparing Mean of the First time of Receiving Additional Analgesic Drug After Surgery in Both IV and Rectal Acetaminophen Receiving Groups Separated by Gender and Age

Variable	No.	Mean	SD	t value	P value
**Gender**					
**Boy**				1.54	0.136
IV acetaminophen	23	3.26	4.87		
Rectal acetaminophen	16	6.62	7.71		
**Girl**				1.1	0.279
IV acetaminophen	20	4.45	4.29		
Rectal acetaminophen	11	2.54	5.12		
**Age Group, y**					
**Below 5**				0.75	0.374
IV acetaminophen	3	1	0		
Rectal acetaminophen	5	4.4	7.6		
**10-5**				0.865	0.391
IV acetaminophen	37	3.94	4.67		
Rectal acetaminophen	21	5.28	7.11		
**Above 10**				0.569	0.626
IV acetaminophen	3	5	6.08		
Rectal acetaminophen	1	1	0		
**Below 25**				0.808	0.42
IV acetaminophen	19	4.94	6.07		
Rectal acetaminophen	15	3.26	4.94		
**Weight Category, kg**					
**25-35**				2.02	0.067
IV acetaminophen	11	2.63	2.2		
Rectal acetaminophen	11	7.63	7.9		
**Above 35**				0.631	0.54
IV acetaminophen	13	3.15	3.28		
Rectal acetaminophen	1	1	0		

## 5. Discussion

This research studied and compared the duration of analgesic effects of two forms of rectal and IV acetaminophen following adenotonsillectomy. The findings showed no significant difference between children conditions in two groups when entering the recovery. Furthermore, a significant relationship was observed between pain in patients of both groups on 4th and 6th hours after surgery indicating that pain was considerably less in rectal than that in intravenous acetaminophen group. Additionally, the need for analgesic medication (CHIPPS values of more than 4) in IV acetaminophen group was significantly higher.

In a study on 50 children, aged between 2-5 years old undergoing adenotonsillectomy, Capici et al. found that receiving the first dose of additional analgesic was later in intravenous group, which was different from the findings of the present work. Also, Capici et al. noticed that few people from both groups received more additional analgesic medication in the first postoperative 6 hours compared to 6 to 10 hours after the operation; while such significant difference was not observed in the present study (1).

Hosseini Jahromi et al. conducted a study in which 120 patients were divided into four groups receiving following drugs following tonsillectomy: lidocaine spray, morphine spray, ketamine spray, and normal saline spray. FLACC pain scale was evaluated during recovery period. The results showed that lidocaine spray had the best pain controlling effect in the early recovery, but after 40 minutes, ketamine and morphine sprays were more effective than lidocaine spray (8). In our study, post-operative pain in rectal acetaminophen group was less than IV acetaminophen group.

In another study on 75 pediatric patients who were scheduled for tonsillectomy, Javid M et al. compared IV and SC ketamine, with placebo. They concluded that in ketamine group, pain score and analgesic consumption were significantly lower (9).

In Boroumand P et al. RCT (randomized control trial) 104 pediatric patients were scheduled for tonsillectomy, advised acetaminophen plus honey or acetaminophen plus placebo. They observed that post-operative honey administration reduced post-operative pain in patients (10).

In a study on 60 children aged 1-10 years old who underwent minor abdominal surgeries, Heshmati et al. compared rectal acetaminophen with intravenous pethidine for postoperative pain and reported that the frequency of postoperative pain in rectal acetaminophen group was less than that in intravenous pethidine receiving group (12). This result was similar to that of the present work.

Alhashemi et al. randomly divided 80 children undergoing tonsillectomy into two groups receiving intramuscular pethidine or intravenous acetaminophen. A higher percentage of patients who received intravenous acetaminophen needed postoperative analgesic medication compared to the ones who received intramuscular pethidine (13). This finding was in agreement with conclusions of the present study on intravenous acetaminophen.

Atef et al. conducted a study in which patients undergoing tonsillectomy were divided into two groups receiving intravenous acetaminophen and placebo (normal saline), and found that the need for analgesic medication in the saline group (71%) was higher than that in intravenous acetaminophen group (0%); accordingly, during 24 hour of patients' follow-up, the need for additional analgesics in the placebo group was 2.2 dose compared to 0.5 in IV acetaminophen group (14).

To sum up considering the results of the present study, the difference between duration of analgesia caused by intravenous and rectal forms of acetaminophen in controlling postoperative pain could be probably due to the time difference between the peak plasma concentrations of these two drugs. Slower absorption of rectal acetaminophen (that lasts about 35-45 min), despite of the first hepatic passage, causes this form of drug to reach its peak plasma concentration after about 2-3 h (15). In contrast, IV acetaminophen reaches its maximum concentration after 15-20 min (16). As a result, analgesic effects of rectal acetaminophen last longer.

Altogether, according to the results obtained from this study, rate of post-adenotonsillectomy pain in 4th and 6th hours after the surgery in rectal acetaminophen group was less than that in the IV group, and the time for consumption the first additional painkiller in rectal group was delayed. Also, the need for postoperative additional analgesic medication in the rectal acetaminophen group was evidently less than that in IV acetaminophen group.
